# The Status of Women in Emergency Ultrasound Fellowships: A Potential Light for the Future of Gender Inclusion in Academic Medicine

**DOI:** 10.7759/cureus.28701

**Published:** 2022-09-02

**Authors:** Josie Acuña, Elaine Situ-LaCasse, Adrienne A Yarnish, Richard Amini, Neil L McNinch, Srikar Adhikari

**Affiliations:** 1 Emergency Medicine, University of Arizona, Tucson, USA; 2 Statistics, McNinch Biostats, LLC, Kent, USA

**Keywords:** ultrasonography, ultrasound, education, diversity, gender, fellowship, emergency medicine

## Abstract

Background: The objective of this study is to investigate gender differences in the percentage of men and women who have completed an Emergency Ultrasound (EUS) fellowship over a four-year period. Secondary objectives of this study include evaluation of the career paths and accomplishments of women who have recently completed an EUS fellowship. We will also be assessing program characteristics as reported by the program directors.

Methods: This was an online survey of all EUS fellowship programs in the United States. Programs were excluded if they were not in existence before July 2020. The survey took place between September 16, 2021, and December 5, 2021. The study was approved by the appropriate institutional review board. Emergency Ultrasound fellowship programs and their respective program directors were identified from a list of participating programs created by the Society of Clinical Ultrasound Fellowships. The survey questions were divided into the following categories: program demographics, questions regarding the program's recent fellowship classes, and questions relating to the program director’s perspective on gender and fellowship.

Results: This study utilized a convenience sample, from a roster of 109 programs, with a response rate of 67% by program directors. There was no significant difference in the percentage of men and women who have completed an EUS fellowship over a four-year period. No graduates who identified as transgender or non-binary/gender non-conform were reported. The majority of female fellows (65%) completed a research/scholarly project during their fellowship year (65%) and have held or currently hold a leadership position at their institution (60.3%). When program directors were asked if they felt women were equally represented in EUS fellowship programs, 24 (34.3%) respondents said yes, 18 (25.7%) said no, and 28 (40%) were not sure. When asked if they felt there were barriers that specifically prevented women from pursuing a fellowship, 28 (40%) said yes, 24 (34.3%) said no, and 18 (25.7%) were not sure.

Conclusion: There does not exist a significant difference in the percentage of males and females who have completed an EUS fellowship over a four-year period. Completion of an EUS fellowship may provide women the opportunity to participate in research and receive awards for their accomplishments. It may also serve as a pipeline to academic medicine and leadership roles.

## Introduction

Despite the progress that is being made, significant gender differences continue to persist in academic emergency medicine (EM). In 2021, Association of American Medical Colleges (AAMC) data demonstrated that only 38.5% of EM academic faculty were women [1]. Another study in 2019 found that of all attending academic EM physicians in the United States (US) with medical school faculty appointments, only 28% were women [2]. In EM residencies, women make up only 36% of current residents. This is in spite of the fact that women made up 48% of US medical school graduates [3,4]. These observations suggest that there is likely a myriad of causes starting in early medical training that lead to the disproportionate number of women in academic EM. One possibility is that perhaps there is a lack of women entering fellowship training after residency. Fellowship training, in general, affords graduating residents the unique opportunity to work at an academic center, often directly after fellowship, as their additional training increases their marketability in academia. Completion of one of the several EM-based fellowships affords a variety of career pathways, including education, research, and administration. Should there be a paucity of women entering fellowship training it may naturally lead to less exposure to and fewer opportunities to engage in academic EM.

The number of Emergency Ultrasound (EUS) fellowship programs and applicants has grown considerably over the recent years. This is a survey of fellowship program directors at all EUS programs in the United States. The objective of this study is to investigate the possible existence of gender differences in the percentage of men and women who have completed an EUS fellowship over a four-year period. Secondary objectives of this study include further evaluation of the career paths and accomplishments of women who have recently completed an EUS fellowship and determine what proportion have pursued a career in academic medicine. In addition, we evaluated program characteristics to assess how this might impact women in academic medicine. With the knowledge that this survey can provide, we can inform the process to improve the recruitment and retention of women in EUS training and academic EM. This article was previously presented as a meeting abstract at the 2022 SAEM Annual Meeting on May 11, 2022.

## Materials and methods

This was an online survey of all EUS fellowship programs in the US. Programs were excluded if they were not in existence before July 2020. The survey took place between September 16, 2021, and December 5, 2021. No incentives were offered. The study was approved by the appropriate institutional review board. The survey was administered to EUS fellowship program directors in the US. Emergency Ultrasound fellowship programs and their respective program directors were identified from a list of participating programs created by the Society of Clinical Ultrasound Fellowships (SCUF) [5]. A total of 109 programs were identified. After compiling a list of all Emergency Ultrasound fellowship programs, the SCUF online directory was used to obtain email addresses for the fellowship program directors. In instances where the program director's email address was not listed or later found to be inaccurate, we went directly to the fellowship program's website to obtain a valid email address. Emails were initially sent with an introductory letter and a link to the online survey hosted by Qualtrics (Qualtrics International Inc, Seattle, WA). Each survey was assigned a random identifier that was used to track the successful completion of the survey by a program. For non-responders, as determined by the survey host, an electronic reminder to complete the survey was sent four weeks after the initial email.

The final survey consisted of 12 multiple-choice and list-type questions, which were divided into the following categories: program demographics, questions regarding the program's recent fellowship classes, and questions relating to the program director’s perspective on gender and fellowship (Appendix 1). The survey instrument was reviewed by a panel of three EM physicians and subsequently pilot tested on three past or current EUS fellowship program directors. No sample size was calculated, as the entire eligible population in the database was included.

Analysis was descriptive in nature, comprising distribution-based summary statistics, in addition to inferential statistics to assess whether there was a gender difference in the total number of fellows over the four-year period via conduct of the sign test. A repeated measures mixed models analysis with restricted maximum likelihood (REML) estimation and unstructured covariance was used to assess for potential changes over time in the percentage of female fellows, as well as in the percentage of female fellows who completed fellowship, received awards, took academic positions, or assumed leadership positions. For significant findings only, post hoc pairwise comparisons were evaluated without type I error rate adjustments due to the initial, exploratory nature of this study. All analyses were conducted using STATA/BE 17.0 (R) (StataCorp, College Station, TX, USA) with results interpreted at a type I error rate of alpha = 0.05 level of statistical significance.

## Results

Program demographics

This study utilized a convenience sample, from a roster of 109 programs, with 73 partial or complete responses (67%) by program directors. Of the 73 program directors who responded, 29 were identified as females and 45 were identified as males. Of the 73 programs represented, the mean (SD) number of faculty was 5.0 (2.9), with a mean (SD) percentage of female faculty of 39.2 (26.5). The mean percentage of female fellows varied between 39.3% and 50.2% during the years represented by the survey. Summary statistics are described in Table 1. More than one-half of the respondents would describe their department as very inclusive (n = 39, 53.4%), while 43 (58.9%) indicated that gender was not a consideration during recruitment. The majority of programs (n = 45, 61.6%) stated that gender was not a consideration when creating their EUS fellowship rank list.

**Table 1:** Emergency Ultrasound Fellowship Program Demographics

Overall number of responses = 75	Number of responses (%)
Geographic location	
Northeast	25 (34.3)
South	22 (30.1)
Midwest	14 (19.2)
West	12 (16.4)
Program director’s perceived degree of inclusiveness in their department	
Very uninclusive	0 (0)
Somewhat uninclusive	3 (4.1)
Neither inclusive nor uninclusive	3 (4.1)
Somewhat inclusive	28 (38.3)
Very inclusive	39 (53.4)
Number of programs that consider gender when recruiting fellows for ultrasound fellowship	
Yes	30 (41.1)
No	43 (58.9)

Summary of fellowship classes

From the responding programs, 146 female fellows were reported to have graduated from EUS fellowships from July 2017 through June 2021. No graduates who identified as transgender or non-binary/gender non-conform were reported. There was no significant difference in the total number of men and women who have completed an EUS fellowship over a four-year period (p-value = 0.130). The median (interquartile range (IQR)) for males is 2.0 (1-4) and for females 2.0 (0-3). Accomplishments by female fellows are summarized in Table 2. Notably, the majority of female fellows were reported to have completed a research/scholarly project during their fellowship year, and the majority have held or currently hold a leadership position at their hospital/institution.

**Table 2:** Accomplishments by Female Fellows

Overall number of responses = 146	Number of responses (%)
Number of female fellows that took a position in academic medicine	66 (45.2)
Number of female fellows that completed a research/scholarly project during fellowship	95 (65)
Number of female fellows that received an award or recognition for work completed	21 (14.4)
Number of female fellows that have held or currently hold a leadership position at their hospital/institution	88 (60.3)
Number of female fellows that have held or currently hold a leadership position nationally	15 (10.3)

An analysis of changes over time in the percentage of female fellows and the percentage of female fellows with particular accomplishments (awards/appointments) was conducted. A statistically significant result was noted for percentage of female fellows holding national positions of leadership (p-value = 0.038), with post hoc differences noted between 2018 and 2019 (p-value = 0.028; mean of 7.4% in 2018 vs mean of 20.4% in 2019), as well as 2020 and 2021 versus 2019 (p-value = 0.015 for each; mean of 20.4% in 2019 vs 0% in 2020 and 13.5% in 2021).

Program director's perspective

Emergency ultrasound program directors were surveyed on their perceptions regarding the status of gender issues in fellowships. When asked if they felt women were equally represented in EUS fellowship programs, 24 (34.3%) respondents said yes, 18 (25.7%) said no, and 28 (40%) said they were not sure. When asked if overall they felt women were equally represented in all EM fellowships, 16 (22.9%) program directors said yes, 24 (34.3%) said no, and 30 (42.8%) said they were not sure.

Program directors were asked if they felt that there were any barriers that specifically prevented women from pursuing a fellowship. Of the responding programs, 28 (40%) said yes, 24 (34.3%) said no, and 18 (25.7%) were not sure. For those respondents who indicated yes, they were further prompted to indicate from a list which barriers they felt deterred women from applying and completing a fellowship program (Table 3).

**Table 3:** Significant Barriers That Deter Women From Applying and Completing a Fellowship Program According to the Emergency Ultrasound Fellowship Directors

Potential barrier	Number of programs that indicated the barrier was significant (%)
Length of time of fellowship program	6 (21.4)
Fellow salary	9 (32.1)
Concerns that fellowship will delay other significant life events	19 (67.9)
Lack of mentorship on pursuing fellowship training and choosing a program	12 (42.9)
Concerns about returning to academic culture after working as a physician in non-academic setting	4 (14.3)
Lack of confidence regarding skills and knowledge	7 (25.0)
Availability of jobs after completing fellowship	6 (21.4)
Concern that ultrasound fellowship graduates have to stay in academics	7 (25)
Lack of equal opportunities for future career success	10 (35.7)
None of the above	2 (7.1)

## Discussion

Significant gender differences continue to persist in EM. Women continue to comprise the minority of academic EM physicians in the US. Yet, women make up nearly half of the US medical school classes [2-4]. This suggests that somewhere along the training and early career, we see a drop in women bound for a lasting career in academic medicine. A significant proportion of physicians who complete a fellowship after their EM training have a high likelihood of entering academic EM. Perhaps, the promotion of a fellowship pathway can lead to more women who choose to continue into academic EM. In keeping with this, we suggest that one potential reason for the disparity of women in academic EM is the difference in the number of women completing the fellowship, compared to men. This study specifically evaluated EUS fellowships. We studied potential gender differences in EUS fellowship classes over a four-year period. While the gender distributions appeared to be slightly different when comparing male and female fellows, the study sample demonstrated that there is no evidence of a significant gender difference. While this is excellent news for ultrasound in particular as a specialty, these data alone are limited in that we are only looking at a four-year period. It would be interesting to compare our data to previous years, and similarly, it may be relevant to track this same cohort again four to five years from the time of data collection to see their ultimate career choice. This also leads us to further question: How does the representation of women in EUS fellowship compare to other EM-based fellowships? And if the gender gap among EM-based fellowships has closed and representation is improving, where are the losses in retention coming from?

While the main objective of this study was to determine whether a gender difference existed in the representation of women versus women in EUS fellowships, we found that the strength of this study was actually in the descriptive statistics relating to the questions aimed at our secondary objectives. Based on our study, 45.2% of female EUS fellowship graduates choose an academic medicine position. This number is promising given the previously mentioned study by Bennett et al., where women made up only 28% of those EM physicians with medical school faculty appointments [2]. In our study, it was reported that over half of the female graduates (60.3%) have held or currently hold a leadership position in their current institution and over 10% have held or currently hold a national leadership position. It was excellent to see how many female graduates have held leadership positions after only several years from completing the fellowship. This is likely secondary to skills gained or augmented during fellowship training, such as completing scholarly projects. Such leadership skills and experience gained during the fellowship create more marketable candidates for subsequent leadership positions and promotion. A study by Goldflam et al. found that EUS fellowship graduates took a variety of leadership positions directly out of training [6]. While many of these were ultrasound based, such as ultrasound division directors and fellowship directors, graduates who also took EUS fellowship also received non-ultrasound-related leadership positions such as medical student clerkship director and associate residency director. Early leadership positions are important when it comes to promoting and pivoting to more desirable roles. Female academic EM physicians still fall behind as a whole when it comes to climbing the ranks and obtaining coveted leadership positions. Female academic EM physicians are less likely to hold the rank of associate or full professors compared to male physicians [2]. Additionally, the proportion of female chairs in EM has yet to exceed 13%. The proportion of women holding department chair positions in EM was 11.3% in 2020 [7]. It is clear that the long-held belief that increasing the number of women in medical schools and EM residencies would lead to more women in academic EM and leadership positions has yet to be proven true [8]. As such, we should be careful to not be as easily pacified that we appear to have bridged the gap in gender representation among EUS fellowship graduates.

Other findings in this study support our hypothesis that completion of an EUS fellowship provides some momentum for women’s careers, particularly in academic medicine. The majority of female fellows were reported to have completed a research/scholarly project during their fellowship year. Such experience is valuable to graduates, particularly those who pursue a career in academics as many academic tracks have promotion processes with research requirements. It may be reasonable to suggest that similar findings would translate to other EM-based fellowships as well, as many incorporate research skills into their curriculum. A small group of female graduates (14.4%) also received awards or recognition for work completed during their fellowship training. A recent study compared the gender distribution of national awards in emergency medicine to the proportion of women in the emergency medicine workforce over a 20-year period [9]. While it was determined that the gender gap in award winners had decreased over time, women represented a smaller proportion of award winners than men when compared with the national proportion of women in academic EM. Specifically, it was noted that women were least likely to receive clinical and leadership awards. In our study, it was not a surprise to find that female graduates have received recognition for their work despite EUS training only being a year. For many, an EUS fellowship year is a busy time, filled with opportunities to teach, research, and collaborate on projects. Program directors often act as mentors and sponsors for their fellows, helping them to gain visibility by promoting their accomplishments and nominating them for awards. These relationships often continue long after fellows have graduated. As the authors Fang et al. poignantly described, honoring all physicians equitably is key to ensuring their longevity and signifies not only inclusion but also value [9].

Another component of this study was to survey EUS fellowship program directors on their perceptions regarding the current status of gender issues in fellowships. There were varied responses to questions regarding female representation in EM-based fellowships. Many indicated that they were unsure if they felt women were equally represented in EUS fellowship programs which shows that there is a knowledge gap on the level of program leaders. This is an opportunity for the education of our field’s leaders, and our study assists with bridging that gap in the literature. Only a minority of directors felt that women were equally represented in EUS fellowships and all EM-based fellowships. Yet even with this in mind, 41.1% indicated that gender was not a consideration during recruitment and the majority of programs stated that gender was not a consideration when creating their EUS fellowship rank list. Herein lies another opportunity for education, with the goal of changing the perception of female fellows. Program directors were also surveyed on the barriers for women deterring them from pursuing a fellowship. Approximately, a quarter of the program directors (PDs) surveyed believed that women’s concerns that fellowship will delay other significant life events were a key deterrent to pursuing a fellowship. The second most common answer was the lack of mentoring in pursuing a fellowship. These responses reflect some of the most stated barriers to gender parity in academic medicine in general. Female physicians weigh family obligations more heavily than male physicians when making career decisions [10]. In turn, this may affect their decision to pursue more training, as opposed to joining the workforce immediately to better serve their family life financially. With regards to the concerns of lack of mentoring in pursuing a fellowship, it appears that the deficiency of mentors for female physicians begins as early as medical school, where female medical students also cite a lack of mentoring and dissatisfaction with their level of mentoring compared to their male counterparts [11,12].

Limitations

The results of this analysis are considered preliminary and exploratory in nature, suitable for the purpose of planning the direction of future research. Analyses were conducted utilizing parametric tests. Follow-up testing with non-parametric testing was conducted with similar findings; therefore, despite the departure of normality noted in some variables, the results presented are based on the parametric testing. Additional limitations, specifically affecting the repeated measures analysis, include the presence of missing data, due to rare instances in which a survey response was incomplete. Finally, the use of a non-probability roster for survey administration, despite a good response rate, and without an a priori sample size analysis for testing a specific hypothesis, may limit the generalizability of the results.

This study only looked at EUS fellowship programs. Although not exhaustive, it serves as a pilot study for a much larger study evaluating gender differences among all emergency medicine fellowships. It is unclear how easily we can generalize the results of this study to other EM-based fellowships. One limitation of this study is the introduction of recall bias as a result of surveying program directors as opposed to fellowship graduates. We attempted to limit the extent of recall bias by limiting the survey questions to pertain to only the past four years of graduates. Despite a shorter timeframe, recall bias will still certainly exist. Unfortunately, it was not feasible to obtain reliable contact information for all fellowship graduates over the 109 programs over a four-year period. To our knowledge, the contact information of prior fellows is not maintained or readily accessible through the SCUF website. With regard to the program directors, a question arises as to whether their gender identity affected how they responded to some of the survey questions. As the survey responses were de-identified once received, we are unable to retrospectively analyze these data. Future studies could benefit from focusing on the program director’s personal characteristics.

One component of this study was to survey program directors on what they perceived to be barriers that deter women from applying and completing a fellowship training. Two PDs stated “None of the above” for the list of barriers deterring women from pursuing the fellowships. We could have had a write-in section for participants to list specific barriers. We attempted to make this list complete by piloting the survey and asking for suggestions, but it appears that it may not be fully inclusive of all barriers.

## Conclusions

There does not exist a significant difference in the percentage of males and females who have completed an EUS fellowship over a four-year period. Completion of an EUS fellowship affords women the opportunity to participate in research and receive awards for their accomplishments. It may also serve as a pipeline to positions in academic medicine and leadership roles. This study assists in better understanding of the larger issue of gender disparity in medicine. The findings further highlight the need for continued work in the areas of education, change in culture, and support for female physicians. The results of this survey can help to inform the process to improve the recruitment and retention of women in EUS training and academic EM.

## Appendices

APPENDIX 1: Survey Questions

Question 1: What is the geographic location of your program?

Northeast (CT, ME, MA, NH, NJ, NY, PA, RI, VT)

West (AK, AZ, CA, CO, HI, ID, NM, OR, MT, UT, NV, WA, WY)

South (AL, AR, DE, DC, FL, GA, KY, LA, MD, MS, NC, OK, SC, TX, TN, VA, WV)

Midwest (IN, IL, MI, OH, WI, IO, KS, MN, MO, NE, ND, SD)

Question 2: With regard to diversity and inclusion, how would you characterize the climate in your department?

Very inclusive (for example, active recruitment of diverse faculty, regular education on diversity and inclusion)  

Somewhat inclusive

Neither inclusive nor uninclusive

Somewhat uninclusive

Very uninclusive (for example, notable lack of diversity among staff, hostility toward initiatives to promote diversity and inclusion)

Question 3: How many current ultrasound fellowship-trained faculty (including pediatric ultrasound fellowship) in your emergency department identify as the following:

Male:                                                                                       

Female:                                                                                   

Transgender or Non-binary/gender non-conforming:            

Unknown:                                                                              

Total:                                                                                   

Question 4: Is gender a consideration when recruiting fellows for your ultrasound fellowship program?

Yes

No

Question 5: Is gender a consideration when creating your rank list for your ultrasound fellowship program?

Yes

No

Question 6: For the academic year 2017/2018, how many of your Emergency Medicine-trained ultrasound fellows identified as the following: If your program did not exist or did not have fellows this year, choose "0" for all sections.
 

Male:                                                                                       _______ 

Female:                                                                                   _______ 

Transgender or Non-binary/gender non-conforming:         _______ 

Unknown:                                                                               _______

Total:                                                                                      _______

Q6a. Of the female fellows that completed fellowship in 2018, how many:

Took a position in academic medicine?

0

1

2

3 or more

Completed a research project during fellowship?

0

1

2

3 or more

Received an award or recognition for work completed during fellowship?

0

1

2

3 or more

Have held or currently hold a leadership position at their hospital/institution?

0

1

2

3 or more

Have held or currently hold a leadership position nationally?

0

1

2

3 or more

Question 7: For the academic year 2018/2019, how many of your Emergency Medicine-trained ultrasound fellows identified as the following:

If your program did not exist or did not have fellows this year, choose "0" for all sections.

Male:                                                                                       _______ 

Female:                                                                                   _______ 

Transgender or Non-binary/gender non-conforming:            _______ 

Unknown:                                                                               _______

Total:                                                                                      _______

Question 7a: Of the female fellows that completed fellowship in 2019, how many:

Took a position in academic medicine?

0

1

2

3 or more

Completed a research project during fellowship?

0

1

2

3 or more

Received an award or recognition for work completed during fellowship?

0

1

2

3 or more

Have held or currently hold a leadership position at their hospital/institution?

0

1

2

3 or more

Have held or currently hold a leadership position nationally?

0

1

2

3 or more

Question 8: For the academic year 2019/2020, how many of your Emergency Medicine-trained ultrasound fellows identified as the following: If your program did not exist or did not have fellows this year, choose "0" for all sections.

Male:                                                                                       _______ 

Female:                                                                                   _______ 

Transgender or Non-binary/gender non-conforming:            _______ 

Unknown:                                                                               _______

Total:                                                                                      _______

Q8a. Of the female fellows that completed fellowship in 2020, how many:

Took a position in academic medicine?

0

1

2

3 or more

Completed a research project during fellowship?

0

1

2

3 or more

Received an award or recognition for work completed during fellowship?

0

1

2

3 or more

Have held or currently hold a leadership position at their hospital/institution?

0

1

2

3 or more

Have held or currently hold a leadership position nationally?

0

1

2

3 or more

Question 9: For the academic year 2020/2021, how many of your fellows identified as the following: If your program did not exist or did not have fellows this year, choose "0" for all sections.

Male:                                                                                       _______ 

Female:                                                                                   _______ 

Transgender or Non-binary/gender non-conforming:            _______ 

Unknown:                                                                               _______

Total:                                                                                      _______

Question 9a: Of the female fellows that completed fellowship in 2021, how many:

Took a position in academic medicine?

0

1

2

3 or more

Completed a research project during fellowship?

0

1

2

3 or more

Received an award or recognition for work completed during fellowship?

0

1

2

3 or more

Have held or currently hold a leadership position at their hospital/institution?

0

1

2

3 or more

Have held or currently hold a leadership position nationally?

0

1

2

3 or more

Question 10: Do you think women are equally represented in ultrasound fellowship programs?

Yes

No

Not Sure

Question 11: Overall, do you think women are equally represented in all Emergency Medicine fellowships?

Yes  

No

Not sure

Question 12: Do you think that there are barriers that specifically prevent women from pursuing fellowship?

Yes

No

Not Sure  

If yes to previous question:

Question 12a: Which of the following do you consider a significant barrier that deters women from applying and completing a fellowship program (Check all that apply):

▢ Length of time of fellowship program

▢ Fellow Salary

▢ Concerns that fellowship will delay other significant life events  

▢ Lack of mentorship on pursuing fellowship training and choosing a program

▢ Concerns about returning to academic culture after working as a physician in non-academic setting

▢ Lack of confidence regarding skills and knowledge

▢ Availability of jobs after completing fellowship

▢ Concern that ultrasound fellowship graduates have to stay in academics

▢ Lack of equal opportunities for future career success

▢ None of the above  
